# A Systematic Review of the Prognostic Significance of the Body Mass Index in Idiopathic Pulmonary Fibrosis

**DOI:** 10.3390/jcm12020498

**Published:** 2023-01-07

**Authors:** Angelo Zinellu, Ciriaco Carru, Pietro Pirina, Alessandro G. Fois, Arduino A. Mangoni

**Affiliations:** 1 Department of Biomedical Sciences, University of Sassari, 07100 Sassari, Italy; 2 Quality Control Unit, University Hospital of Sassari (AOU), 07100 Sassari, Italy; 3 Department of Medical, Surgical and Experimental Sciences, University of Sassari, 07100 Sassari, Italy; 4 Clinical and Interventional Pneumology, University Hospital Sassari (AOU), 07100 Sassari, Italy; 5 Discipline of Clinical Pharmacology, College of Medicine and Public Health, Flinders University, Bedford Park, SA 5042, Australia; 6 Department of Clinical Pharmacology, Flinders Medical Centre, Southern Adelaide Local Health Network, Bedford Park, SA 5042, Australia

**Keywords:** body mass index, idiopathic pulmonary fibrosis, biomarker, adverse outcomes, mortality, hospitalization, disease progression, prognosis

## Abstract

The identification of novel prognostic biomarkers might enhance individualized management strategies in patients with idiopathic pulmonary fibrosis (IPF). Although several patient characteristics are currently used to predict outcomes, the prognostic significance of the body mass index (BMI), a surrogate measure of excess fat mass, has not been specifically investigated until recently. We systematically searched PubMed, Web of Science, and Scopus, from inception to July 2022, for studies investigating associations between the BMI and clinical endpoints in IPF. The Joanna Briggs Institute Critical Appraisal Checklist was used to assess the risk of bias. The PRISMA 2020 statement on the reporting of systematic reviews was followed. Thirty-six studies were identified (9958 IPF patients, low risk of bias in 20), of which 26 were published over the last five years. Significant associations between lower BMI values and adverse outcomes were reported in 10 out of 21 studies on mortality, four out of six studies on disease progression or hospitalization, and two out of three studies on nintedanib tolerability. In contrast, 10 out of 11 studies did not report any significant association between the BMI and disease exacerbation. Our systematic review suggests that the BMI might be useful to predict mortality, disease progression, hospitalization, and treatment-related toxicity in IPF (PROSPERO registration number: CRD42022353363).

## 1. Introduction

Idiopathic pulmonary fibrosis (IPF) is clinically characterized by an insidious decline in lung function, which generally leads to respiratory failure and death within four years of diagnosis [1]. However, significant inter-individual variability exists in disease progression. This variability is at least partly related to the frequency of disease exacerbations and the presence of specific comorbid conditions [2,3,4,5]. Several patient characteristics, as well as measures of lung function, have also been shown to predict survival and other relevant outcomes, e.g., disease progression and exacerbation, in this patient group. In particular, advancing age, male sex, lower values of forced vital capacity (FVC) and diffusing capacity of carbon monoxide (D_LCO_) percentage predicted at baseline and during follow-up, severe dyspnea, supplemental oxygen requirement, lower distance walked during the six-minute walk test (6MWT), and greater fibrotic burden on high resolution computed tomography (HRCT) are currently used as prognostic markers in IPF. Their use is typically combined in validated clinical prediction models, such as the gender-age-physiology (GAP) model, the longitudinal GAP model, and the composite physiologic model [1,2,6,7,8,9,10]. However, the predictive capacity of available tools could be potentially improved following the identification of additional biomarkers.

There is an intense research focus on determining the prognostic role of several circulating biomarkers, e.g., small molecules and peptides, that are involved in pathways thought to play a critical pathophysiological role in IPF. However, the widespread clinical use of such biomarkers is likely to be curtailed by the highly specific and expensive analytical techniques and facilities often required for their determination, particularly in less developed countries [11,12,13,14]. An alternative approach in the quest for novel prognostic biomarkers consists of the identification of alternative clinical characteristics that are routinely assessed in patients with IPF. In this context, an increasing number of studies have investigated the prognostic role of the body mass index (BMI), a surrogate marker of body fatness routinely used in the risk stratification of patients with cardiovascular disease, diabetes, and other metabolic disorders [15,16,17,18], in IPF. Therefore, we sought to critically appraise the available evidence regarding the prognostic significance of the BMI in IPF by conducting a systematic review of studies reporting associations between baseline BMI values and their temporal changes, clinical outcomes, and other relevant clinical parameters in this patient group.

## 2. Materials and Methods

We systematically searched the electronic databases PubMed, Web of Science, and Scopus for articles published between inception and 15 July 2022, using the following terms and their combination: “BMI” or “body mass index” and “IPF” or “idiopathic pulmonary fibrosis”. Two investigators independently reviewed the abstracts and, if relevant, the full articles. The citation lists of these articles were also hand-searched to identify additional studies. The inclusion criteria for selection were: (a) description of associations between the BMI and clinical outcomes or other relevant clinical parameters in observational and intervention studies in patients with IPF; (b) full-text available, and (c) English language used. The following data were extracted from each study and transferred into an electronic spreadsheet: age, sex, year of publication, country where the study was conducted, study design (observational, prospective vs. retrospective, or randomized controlled study), sample size, criteria used for the diagnosis of IPF, pharmacological treatment for IPF, main comorbid conditions, clinical endpoints assessed, baseline BMI, whether the BMI was assessed as a continuous variable or using cut-off values, results of multivariate Cox regression with details of analyzed confounders, and other univariate associations between the BMI and relevant clinical variables. The Joanna Briggs Institute Critical Appraisal Checklist was used to assess the risk of bias [19]. The PRISMA 2020 statement on the reporting of systematic reviews was followed (Supplementary Tables S1 and S2) [20]. The protocol was registered in the International Prospective Register of Systematic Reviews (PROSPERO, CRD42022353363).

## 3. Results

### 3.1. Study Selection

**Figure 1:**
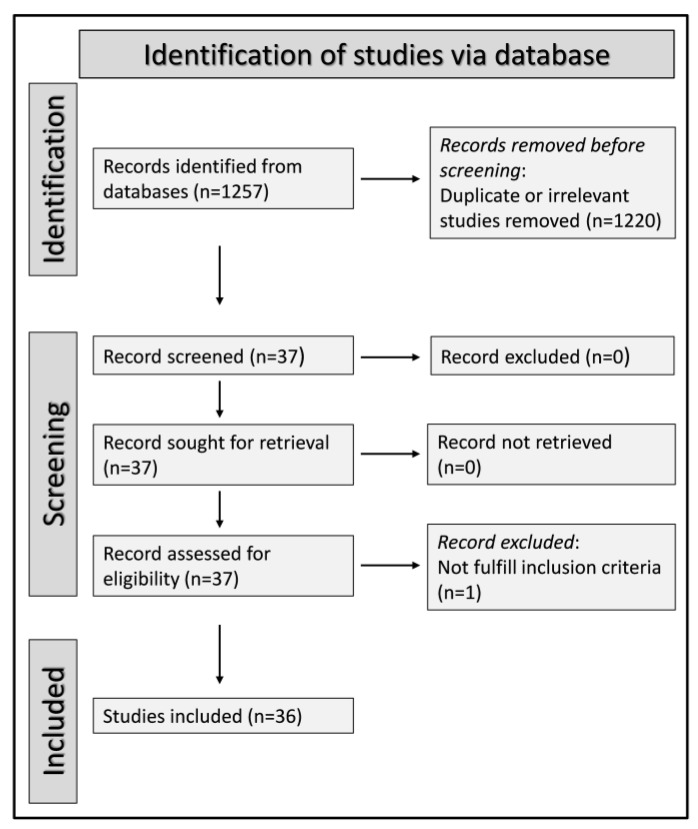
PRISMA 2020 flow diagram.

A total of 1257 articles were initially identified. Among them, 1220 were excluded because they were either duplicates or irrelevant. After a full-text review of the remaining 37 articles, one was further excluded because it did not fulfill the inclusion criteria, thus leaving 36 studies (9958 IPF patients, 78% males) for final analysis [21,22,23,24,25,26,27,28,29,30,31,32,33,34,35,36,37,38,39,40,41,42,43,44,45,46,47,48,49,50,51,52,53,54,55,56] (Figure 1). Fourteen studies were conducted in Japan [22,27,29,30,32,33,34,35,36,37,43,45,50,51], nine in the USA [21,25,31,38,41,46,47,48,49], three in France [44,53,54], three in Italy [26,52,56], three in China [28,39,42], two in South Korea [24,55], one in Ireland [23], and one in Saudi Arabia [40]. The reported clinical endpoints included mortality in 21 studies [21,22,23,24,26,29,30,32,33,37,38,39,40,45,49,50,52,53,54,55,56], disease exacerbation in 11 [22,23,25,27,28,31,34,37,41,46,55], disease progression in five [42,43,44,47,54], hospitalization in three [48,53,54], tolerability to the antifibrotic agent nintedanib in three [35,36,51], and incident pneumothorax in one [32]. Ten studies were prospective [25,26,27,28,30,43,44,46,53,54], while the remaining 26 were retrospective [21,22,23,24,29,31,32,33,34,35,36,37,38,39,40,41,42,45,47,48,49,50,51,52,55,56]. The baseline mean/median BMI values in these studies ranged between 21 and 30 kg/m^2^. Twenty-nine studies assessed the BMI as a continuous variable [21,22,23,25,26,27,28,29,30,31,32,33,34,35,37,39,40,41,43,45,46,47,48,51,52,53,54,55,56], six used cut-off values [24,36,38,42,44,49], and one assessed both [50]. Twenty-six out of 36 studies were published over the last five years [31,32,33,34,35,36,37,38,39,40,41,42,43,44,45,46,47,48,49,50,51,52,53,54,55,56] (Table 1).

### 3.2. Risk of Bias

The risk of bias was assessed as low in 20 studies [21,22,24,29,32,33,35,36,38,39,40,43,45,48,50,51,52,53,55,56] and high in the remaining 16 studies [23,25,26,27,28,30,31,34,37,41,42,44,46,47,49,54] (Table 2).

### 3.3. Results of Individual Studies and Syntheses

#### 3.3.1. Mortality

A significant association between the BMI and mortality was reported in 10 studies, including nine retrospective studies and nine with low risk of bias [21,24,29,38,49,50,52,53,55,56] (Table 1). Alakhras et al. were the first to report a significant relationship between the BMI and survival in 197 IPF patients categorized according to BMI tertiles (<25, 25–30, and >30 kg/m^2^). The bottom tertile (*n* = 46) had a median survival of 3.6 years [1-year survival rate, 76% (95% CI 65 to 90); 3-year survival rate, 54% (95% CI 41 to 70)]. The middle tertile (*n* = 85) had a median survival of 3.8 years [1-year survival rate, 84% (95% CI 76 to 92); 3-year survival rate, 58% (95% CI 48 to 70)]. The upper tertile (*n* = 66) had a median survival of 5.8 years (1-year survival rate, 91% (95% CI 84 to 98); 3-year survival rate, 69% (95% CI 58 to 81%)]. Proportional hazards regression showed a significant, independent, and negative association between the baseline BMI and mortality [21]. Kim et al. reported an independent association between baseline BMI values < 18.5 kg/m^2^ and increased 15-year mortality in 67 IPF patients [24]. Kishaba et al. investigated the impact of changes in BMI during the first year on 12-year mortality. In their analysis, the magnitude of BMI reduction was significantly associated with mortality after adjusting for several confounders, including hospitalization during the first year. Similar associations with 12-year mortality were observed with absolute values of baseline and one-year BMI [29]. Kulkarni et al. also investigated the association between BMI temporal trajectories and one-year transplant or mortality and post-transplant mortality in a discovery cohort (*n* = 131). The quartile with the greatest temporal BMI reduction (>0.68%/month) was independently associated with a higher risk of transplant or death. The association with mortality was maintained after excluding patients undergoing transplant (HR = 2.9, 95% CI 1.6 to 5.2, *p* = 0.0002). In further analysis, patients with temporal BMI reduction >0.68%/month in the year preceding the transplant also had a greater risk of mortality following surgery (HR = 4.6, 95% CI 1.7 to 12.6, *p* = 0.003). The same authors confirmed the presence of an independent association between temporal BMI reduction >0.68%/month and risk of transplant or death in a validation cohort (*n* = 148) [38]. Sangani et al. retrospectively investigated 138 IPF patients categorized as non-obese (BMI < 30 kg/m^2^) and obese (BMI ≥30 kg/m^2^). The usual interstitial pneumonia pattern was less prevalent in the obese group (69% vs. 85%, *p* = 0.007). Significantly lower mortality was observed in this group. A similar trend was also observed when BMI values were analyzed as tertiles (mortality of 20%, 47%, and 75% for BMI values of 25–29.9, 20–24.9, and <20 kg/m^2^, respectively, *p* < 0.001) [49]. Two cohorts receiving antifibrotic treatment with pirfenidone or nintedanib, for a total of 208 IPF patients, were investigated by Suzuki et al. A significant, negative, and independent association was observed with five-year mortality both when considering BMI values as a continuous variable and using a cut-off value of 24.1 kg/m^2^ [50]. Zinellu et al. reported a negative and independent association between the baseline BMI and four-year mortality in a cohort of 82 IPF patients, after adjusting for several confounders including the recently developed aggregate index of systemic inflammation [52,57,58,59,60]. In another prospective cohort study in 153 newly diagnosed IPF patients, Jouneau et al. reported that a lower baseline BMI was independently associated with one-year mortality in multivariate analysis, after adjusting for age, sex, GAP score, and self-evaluation of food intake [53]. Yoo et al. similarly reported that a lower baseline BMI was independently associated with higher three-year mortality in 445 patients with IPF, after adjusting for several confounders including the Charlson comorbidity index, disease progression, and acute exacerbation [55]. Finally, Zinellu et al. investigated 90 IPF patients and reported an independent association between the baseline BMI and four-year mortality, with an area under the curve (AUC) of 0.702 [56]. Incorporating the BMI into a four-domain predictive model (IC4) including the six-minute walking distance, FVC, and D_LCO_ significantly increased the AUC to 0.859 (95% CI 0.770–0.924, *p* < 0.0001) [56].

In contrast, 11 studies, including eight retrospective studies and six with low risk of bias, failed to report a significant association between the BMI and mortality [22,23,26,30,32,33,37,39,40,45,54]. A non-significant association between the BMI and mortality was reported in multivariate analyses in six studies [26,32,33,39,40,45]. Four studies failed to demonstrate a significant association in univariate analysis [22,23,30,37], whereas the remaining study, a post-hoc analysis of five randomized placebo-controlled trials investigating the effects of pirfenidone, interferon-**γ**-1b, and the monoclonal antibody lebrikizumab, did not report a formal statistical analysis of the association between the BMI and one-year mortality [54].

#### 3.3.2. Disease Exacerbation

Only one study reported significant associations between the baseline BMI and risk of disease exacerbation. Kondoh et al. observed an independent and positive association between the baseline BMI and risk of three-year exacerbations in 64 IPF patients [22]. In contrast, no significant associations were reported in the remaining 10 studies, including six retrospective studies and nine with a high risk of bias, all of which reported data from univariate analyses [23,25,27,28,31,34,37,41,46,55].

#### 3.3.3. Disease Progression

Two studies reported a significant impact of the BMI on IPF progression. Fang et al. reported that patients exhibiting disease progression at one year had significantly lower baseline BMI values than those with stable disease. A significant association was also observed with the Kaplan-Meyer log-rank test using a cut-off of ≥25 kg/m^2^ [42]. Similarly, in a post-hoc analysis of a randomized placebo-controlled trial investigating pirfenidone, Ikeda et al. observed that a lower baseline BMI was independently associated with one-year progression. Notably, this association was observed both in the placebo and pirfenidone groups [43]. In contrast, two studies failed to report a significant association with disease progression in univariate analyses [44,47]. In one study, while a significantly greater decline in FVC was observed in patients with BMI < 27 kg/m^2^, no significant BMI-related differences were reported with temporal changes in FVC (% predicted) and St. George’s Respiratory Questionnaire [44]. In a further study, no formal statistical analysis was presented on the association between the baseline BMI and one-year disease progression [54].

#### 3.3.4. Nintedanib Tolerance

Two Japanese studies investigated the potential influence of the BMI on the risk of early discontinuation of treatment with the antifibrotic drug nintedanib, with contrasting results. Ikeda et al. observed that lower baseline BMI values were significantly and independently associated with increased risk of discontinuation in 72 IPF patients [35]. In contrast, no significant association was observed between the baseline BMI and risk of early discontinuation after adjusting for FVC (% predicted) in the study by Uchida et al. involving 78 patients with IPF [51]. In another Japanese study, a BMI of <21.6 kg/m^2^ was independently associated with a tenfold increase in the risk of developing nausea and a threefold increase in the risk of developing diarrhea during nintedanib treatment [36].

#### 3.3.5. Other Clinical Outcomes

Two studies reported a negative association between the BMI at baseline and the risk of hospitalization. Kim et al. observed that a lower BMI was significantly and independently associated with a higher rate of respiratory-related hospitalizations within two years in 1002 IPF patients [48]. Similarly, Jouneau et al. reported that a lower BMI was independently associated with one-year hospitalization in 153 patients with IPF [53]. In another study by Jouneau et al., the associations between BMI tertiles and all-cause hospitalization at one year were not statistically assessed [54]. Finally, Nishimoto et al. reported that lower BMI values at baseline were independently associated with a statistically higher risk of pneumothorax in a retrospective study of 71 IPF patients. In this study, incident pneumothorax was independently associated with increased mortality after adjusting for age, sex, and FVC (% predicted) [32].

## 4. Discussion

In our systematic review, we identified 36 studies assessing the prognostic role of baseline and temporal changes in BMI values in IPF patients receiving a range of immunosuppressive and antifibrotic therapies. Whilst there is currently no evidence of a link between the BMI and a diagnosis of IPF, the available evidence suggests that this routinely assessed surrogate marker of body fatness is a promising predictor of mortality, disease progression, hospitalization, tolerability to specific antifibrotic treatments, and specific complications, i.e., pneumothorax, in this group. In particular, relatively low BMI values at baseline and/or greater temporal declines in BMI are associated with adverse clinical outcomes, barring the risk of disease exacerbation.

The BMI was first described by Quetelet, a Belgian scientist, as an anthropometric index in the nineteenth century under the denomination “social physics” [61]. Following the first publication under its current name in 1972 [62], the BMI has been extensively used in clinical practice and public health screening and intervention programs to categorize people as underweight (<18.5 kg/m^2^), normal weight (≥18.5 and <25.0 kg/m^2^), overweight (≥25.0 and <30.0 kg/m^2^), and obese (≥30.0 kg/m^2^). Although several experts have questioned the physiological significance of the BMI as a reliable indicator of adiposity and excess fat, its use has significantly contributed to the stratification of short- and long-term risks associated with key disease states, e.g., cardiovascular disease, diabetes, and several types of cancer, and to promote lifestyle interventions aimed at reducing this risk both individually and at the population level [15,16,17,18,63]. However, while the health risks associated with relatively higher BMI values are well established, an increasing number of studies over the last decade have reported that individuals with relatively higher BMI and specific overt disease states, e.g., heart failure and cancer, have a more favorable prognosis than those with lower BMI values [64,65]. This phenomenon, known as the “obesity paradox,” has also been described in respiratory conditions such as chronic obstructive pulmonary disease [66,67]. One possible explanation for the putative protective effects of higher BMI values in these conditions and IPF is related to the inherent limitations of this index as a reliable measure of excess fat mass and adiposity. The formula used for its calculation (body weight in kg divided by height in m^2^) does not take into consideration whether changes in body weight are secondary to changes of specific body composition compartments, e.g., fat mass vs. fat-free mass, and/or their distribution, e.g., visceral vs. subcutaneous adiposity [68,69]. Furthermore, a concomitant increase in fat mass (obesity) and a reduction in fat-free mass (sarcopenia) can occur in the same individual. This condition, also known as sarcopenic obesity, is associated with a worse prognosis in disease states such as heart failure and cancer [70,71,72]. Therefore, it is possible that a higher BMI in patients with IPF experiencing a more favorable prognosis is not primarily associated with an increase in fat mass, but rather with an increase in fat-free mass, e.g., muscle mass. This might lead to increased exercise tolerance and cardiorespiratory fitness through increased oxygen consumption via increased muscle diffusion, mitochondrial respiration capacity, and skeletal muscle strength, as already proposed in patients with heart failure [73,74]. This hypothesis is further supported by the results of studies reporting that lower skeletal muscle mass and strength are significantly associated with advanced disease and mortality in patients with IPF [75,76,77]. Furthermore, one study in our systematic review reported a significant and positive association between BMI and the cross-sectional area of elector spine muscles, an imaging parameter used to investigate sarcopenia and cachexia. However, no significant associations were reported with another parameter, muscle attenuation of elector spine muscles [33]. In another study, a significant and positive association was reported between the relative temporal decline in BMI and temporal reduction in the cross-sectional area of elector spine muscles in IPF patients [45]. Another possibility is that interplay between the BMI and clinical outcomes in patients with IPF is modulated by the coexistence of disease states, e.g., heart failure, where an inverse association between BMI values and adverse outcomes has been described [64]. However, this hypothesis requires further investigation as the presence of comorbidities was described in only nine of the studies identified in our systematic review [23,25,29,40,41,42,49,52,53].

It is important to emphasize that several studies failed to report significant associations between the BMI and mortality [22,23,26,30,32,33,37,39,40,45,54] or disease progression [44,47]. Possible reasons for such discrepancies include between study differences in baseline patient characteristics, including severity of IPF, comorbidity burden, ethnicity, and specific treatment received. However, as previously mentioned, information regarding comorbidities was provided in a limited number of studies [23,25,29,40,41,42,49,52,53]. More research is required to investigate possible differences in studies reporting negative findings and to determine whether the prognostic significance of the BMI varies across IPF subgroups.

Another intriguing observation is the possible reduced tolerance to the antifibrotic agent nintedanib in IPF patients with lower BMI reported in two of three studies [35,36,51]. This issue is clinically relevant as the early discontinuation of antifibrotic therapy is associated with worse outcomes in this group [78]. Nintedanib is a relatively fat-soluble drug with a large volume of distribution in humans [79,80]. Assuming that a lower BMI value is secondary, at least partly, to a reduced fat mass, the consequent reduction in the volume of distribution might theoretically lead to higher circulating concentrations of this agent. However, whether this phenomenon accounts for increased risk of toxicity and early treatment discontinuation deserves further study.

In order to establish the prognostic significance of the BMI in IPF, larger and appropriately designed prospective studies are warranted to confirm the findings of our review. Such trials should investigate the predictive capacity of the BMI, singly or in combination with other clinical characteristics and lung function parameters, in IPF patients with a wide range of clinical severity, comorbid status, sarcopenia, and immunosuppressive and antifibrotic treatments. The potential utility of combining the BMI with other parameters in prediction models was recently reported by Zinellu et al. in a study where the incorporation of BMI with 6MWD, FVC, and D_LCO_ significantly increased the AUC for predicting four-year mortality [56].

The strengths of our systematic review include the assessment of a wide range of clinical endpoints as well as the association between the BMI and other relevant patient characteristics, including parameters of lung function and functional capacity. Furthermore, the selected studies investigated Asian, European, and North American patient populations, ensuring some degree of generalization of the findings, and the risk of bias was considered low in the majority of studies (20 out of 36). The limitations of our review include the lack of meta-analytical evaluation given the between study differences in the assessment of the BMI as a continuous variable or cut-off value, baseline variable vs. temporal changes, type of endpoint assessed, and the paucity of details regarding specific comorbidities, markers of muscle mass, and sarcopenia in most studies.

## 5. Conclusions

Our systematic review has shown that the BMI has the potential to be used as an easily measured and inexpensive predictive marker in IPF, particularly for mortality, disease progression, risk of hospitalization, and tolerability to specific therapies. However, prospective, accurately designed studies are warranted to convincingly demonstrate the prognostic utility of this anthropometric parameter and justify its widespread use in the routine management of patients with IPF.

## Figures and Tables

**Table 1:** Characteristics of the studies investigating the association between body mass index and adverse outcomes in idiopathic pulmonary fibrosis.

First Author, Year, Country (Ref)	Study Design	Sample Size Age (Years) M/F	Diagnosis Treatment Endpoint(s)	Baseline BMI (kg/m^2^) BMI Assessment in Cox Model Main Comorbidities	Results of Multivariate Cox Regression Confounders in the Model	Additional Findings
Alakhras M, 2007, USA [21]	R	197 71 137/60	ATS/ERS Colchicine, prednisolone 3-year mortality	28 Continuous variable NR	HR = 0.86, 95% CI 0.79 to 0.94, *p* < 0.001 Sex, diagnosis by open lung biopsy, FVC (% predicted), D_LCO_ (% predicted), O_2_ saturation with exercise	No significant differences between BMI tertiles (<25, ≥25 and <30, and ≥30) in age, sex, smoking status, baseline pulmonary function tests, or recommended treatment at the index visit
Kondoh Y, 2010, Japan [22]	R	74 64 61/13	ATS/ERS Prednisone, cyclophosphamide, azathioprine, cyclosporin 3-year acute exacerbation, 3-year mortality	23 Continuous variable NR	*Acute exacerbation* HR = 1.20, 95% CI 1.03 to 1.40, *p* < 0.001 mMRC scale (2 and above), 10% decline in FVC at 6 months	No significant association between BMI and mortality in univariate Cox regression (HR = 0.97, 95% CI 0.88 to 1.07, *p* = 0.590)
Judge EP, 2012, Ireland [23]	R	55 60 41/14	NR NR Acute exacerbation, 5-year mortality	26 Continuous variable PH	NR	No significant association between BMI and acute exacerbation in univariate Cox regression (HR = 1.043, 95% CI 0.939 to 1.159, *p* = 0.437) No significant association between BMI and mortality in univariate Cox regression (HR = 0.984, 95% CI 0.886 to 1.092, *p* = 0.758)
Kim JH, 2012, South Korea [24]	R	67 70 43/24	ATS/ERS/JRS/ALAT Steroids 15-year mortality	23 BMI < 18.5 NR	HR = 12.085, 95% CI 1.277 to 114.331, *p* = 0.030 Age, sex, FVC <70% predicted, respiratory symptoms at diagnosis, disease progression on CT before 36 months	NR
Lee JS, 2012, USA [25]	P	54 65 42/12	ATS Steroids Acute exacerbation	25 Continuous variable CAD, GERD, OSA, PH	NR	No significant association between BMI and acute exacerbation in univariate Cox regression (OR = 1.04, 95% CI 0.91 to 1.20, *p* = 0.55)
Mura M, 2012, Italy [26]	P	70 67 57/13	ATS NR 3-year mortality	27 Continuous variable NR	No significant associations between BMI and mortality in multivariate analysis (data not reported), mMRC, 6MWD, desaturation during 6MWT, alveolar-arterial O_2_ tension, FVC (% predicted), D_LCO_ (% predicted), HRCT fibrosis score, bronchoalveolar lavage total cell count, concomitant emphysema, CPI	Significant association between BMI and mortality in univariate Cox regression (HR = 0.89, 95% CI 0.80 to 0.98, *p* = 0.01)
Kondoh Y, 2015, Japan [27]	P	267 65 213/54	JRS Pirfenidone Acute exacerbation	24 Continuous variable NR	NR	No significant association between BMI and acute exacerbation in univariate Cox regression (HR = 0.935, 95% CI 0.782 to 1.118, *p* = 0.46)
Cao M, 2016, China [28]	P	62 66 51/11	ATS/ERS/JRS/ALAT NR Acute exacerbation	24 Continuous variable NR	NR	No significant difference in BMI between patients with and without exacerbation (24.1 ± 2.9 vs. 24.6 ± 2.7, *p* = 0.679)
Kishaba T, 2016, Japan [29]	R	65 72 41/24	ATS/ERS/JRS/ALAT Prednisolone, cyclosporin, pirfenidone, nintedanib 12-year mortality	25 Continuous variable (BMI changes during the first year) DM, HT	HR = 1.324, 95% CI 1.045 to 1.676, *p* = 0.02 FVC (% predicted) changes during the first year, hospitalization during the first year	Significant associations between mortality and baseline (HR = 7.708, 95% CI 2.669 to 12.748, *p* = 0.008) and 1-year BMI (HR = 9.058, 95% CI 2.925 to 15.192, *p* = 0.009) in univariate Cox regression
Nishiyama O, 2017, Japan [30]	P	44 72 35/9	ATS/ERS/JRS/ALAT No treatment 4-year mortality	23 Continuous variable NR	NR	No significant association between BMI and mortality in univariate Cox regression (HR = 0.88, 95% CI 0.76 to 1.02, *p* = 0.09). Significant associations between BMI and age (r = −0.33, *p* = 0.03), D_LCO_ (r = 0.50, *p* = 0.0005), 6MWT (r = 0.35, *p* = 0.02), and GAP index (r = −0.42, *p* = 0.003)
Dotan Y, 2018, USA [31]	R	89 66 64/25	ATS/ERS Pirfenidone, nintedanib Acute exacerbation	27 Continuous variable NR	NR	No significant difference in BMI between patients with and without exacerbation (27 ± 5 vs. 28 ± 4, *p* = 0.26)
Nishimoto K, 2018, Japan [32]	R	84 71 74/10	ATS/ERS/JRS/ALAT Prednisolone, cyclophosphamide, cyclosporin, tacrolimus, pirfenidone, nintedanib Pneumothorax, 12-year mortality	22 Continuous variable NR	*Pneumothorax* HR = 0.80, 95% CI 0.67 to 0.94, *p* = 0.008 Extent of reticular abnormalities on HRCT (grade ≥2) *Mortality* HR = 1.01, 95% CI 0.88 to 1.15, *p* = 0.894 Age, sex, FVC (% predicted), pneumothorax, extent of reticular abnormalities (grade ≥2), acute exacerbation	NR
Suzuki Y, 2018, Japan [33]	R	131 69 117/14	ATS/ERS/JRS/ALAT NR 20-year mortality	23 Continuous variable NR	HR = 1.009, 95% CI 0.892 to 1.141, *p* = 0.89 Age, sex, ESM_CSA_, ESM_MA_, FVC (% predicted), FEV_1_/FVC, D_LCO_ (% predicted)	Significant association between BMI and ESM_CSA_ (r = 0.500, *p* < 0.0001). No significant association between BMI and ESM_MA_ (r = 0.01, *p* = 0.90)
Hanaka T, 2019, Japan [34]	R	89 72 74/15	ATS/ERS/JRS/ALAT NR Acute exacerbation	23 Continuous variable NR	NR	No significant difference in median BMI between patients with and without exacerbation (22.9, IQR 21.1–25.8 vs. 22.9, IQR 20.7–24.7, *p* = 0.785)
Ikeda S, 2019, Japan [35]	R	30 72 24/6	ATS/ERS/JRS/ALAT Nintedanib Early nintedanib termination	21 Continuous variable NR	HR = 0.487, 95% CI 0.280 to 0.849, *p* = 0.01 Surfactant protein D, weight loss (grade ≥2) during prior treatment with pirfenidone	Median BMI significantly lower in patients switched from pirfenidone to nintedanib than in patients naïve to pirfenidone (21.0, IQR 19.0–23.6 vs. 23.9, IQR 20.7–26.2, *p* = 0.001)
Kato M, 2019, Japan [36]	R	77 72 65/12	ATS/ERS/JRS/ALAT Nintedanib, prednisolone Nintedanib-induced nausea and diarrhea	23 BMI < 21.6 NR	*Nausea* HR = 10.841, 95% CI 2.644 to 44.448, *p* = 0.001 Performance status, mMRC, GAP index, co-treatment with prednisolone, nintedanib dose *Diarrhea* HR = 3.460, 95% CI 1.044 to 11.467, *p* = 0.04 Performance status, mMRC, GAP index, co-treatment with prednisolone, nintedanib dose	BMI AUC for nausea (0.873, 95% CI 0.784 to 0.962, *p* = 0.001) BMI AUC for diarrhea (0.668, 95% CI 0.502 to 0.834, *p* = 0.036)
Kono M, 2019, Japan [37]	R	96 72 77/19	ATS/ERS/JRS/ALAT Pirfenidone, prednisolone, immunosuppressants Acute exacerbation, 4-year mortality	22 Continuous variable NR	NR	No significant association between BMI and acute exacerbation in univariate Cox regression (HR = 1.096, 95% CI 0.989 to 1.912, *p* = 0.08). No significant association between BMI and mortality in univariate Cox regression (HR = 0.610, 95% CI 0.107 to 3.173, *p* = 0.56)
Kulkarni T (a), 2019, USA [38]	R	131 69 101/30	ATS/ERS/JRS/ALAT NR 1-year transplant or death, mortality post-transplant	30 BMI reduction >0.68%/month NR	*1-year transplant or death* HR = 1.8, 95% CI 1.1 to 3.2, *p* = 0.038 Age, pulmonary function, baseline BMI	Significant association between BMI reduction >0.68%/month pre-transplant and post-transplant mortality in univariate Cox regression (HR = 4.6, 95% CI 1.7 to 12.6, *p* = 0.003). Significant correlation between changes in BMI and changes in serum leptin (r = 0.43, *p* < 0.01) and serum adiponectin (r = −0.33, *p* = 0.04) Lower CD28% in patients with BMI reduction >0.68%/month (*p* = 0.018)
Kulkarni T (b), 2019, USA [38]	R	148 65 100/48	ATS/ERS/JRS/ALAT NR 1-year transplant or death	30 BMI reduction >0.68%/month NR	*1-year transplant or death* HR = 2.5, 95% CI 1.2 to 5.2, *p* = 0.02 Age, pulmonary function, baseline BMI	NR
Li B, 2019, China [39]	R	148 65 108/40	ATS/ERS/JRS/ALAT NR 6-year mortality	24 Continuous variable NR	HR = 0.97, 95% CI 0.89–1.04, *p* = 0.374 FVC (% predicted), serum albumin, serum globulin, serum prealbumin	No significant difference in median BMI between patients with serum prealbumin concentrations <0.2 and ≥0.2 mg/L (24.4, IQR 21.9–26.9 vs. 23.7, IQR 25.4–27.5, *p* = 0.063)
Alhamad EH, 2020, Saudi Arabia [40]	R	212 66 150/62	ATS/ERS/JRS/ALAT Pirfenidone, nintedanib 10-year mortality	27 Continuous variable PH, DM, HT, OP, GORD, CAD	HR = 0.948, 95% CI 0.896–1.003, *p* = 0.06 Acute exacerbation, 6MWT final SpO_2_ <85%, antifibrotic therapy, 6MWTD <300 m, TLC (% predicted), FVC (% predicted)	NR
Dotan Y, 2020, USA [41]	R	89 66 61/28	ATS/ERS/JRS/ALAT NR Acute exacerbation	28 Continuous variable DM, HT, CAD	NR	No significant difference in BMI between patients with and without exacerbation (28 ± 4 vs. 28 ± 4, *p* = 0.28)
Fang C, 2020, China [42]	R	117 64 110/7	ATS/ERS Pirfenidone, prednisone, cyclophosphamide, azathioprine, methotrexate, tacrolimus 1-year disease progression	24 BMI < 25 DM, HT, CAD	NR	Significant difference in BMI between patients with stable disease and those with progressive disease (24.8 ± 2.7 vs. 22.9 ± 3.0, *p* = 0.005). Kaplan-Meyer log-rank test for progression-free survival with BMI ≥ 25 (HR = 2.81, 95% CI 1.03 to 7.68, *p* = 0.044)
Ikeda K, 2020, Japan [43]	P	267 65 213/54	ATS/ERS Pirfenidone, placebo 1-year disease progression	24 Continuous variable NR	*Placebo group* HR = 0.833, 95% CI 0.704 to 0.985, *p* = 0.03 Lowest SpO_2_ during 6MWT, FVC (% predicted) *Pirfenidone group* HR = 0.849, 95% CI 0.723 to 0.998, *p* = 0.046 Smoking status, alveolar-arterial O_2_ difference, FVC (% predicted), surfactant protein D	NR
Jouneau S, 2020, France [44]	P	1,061 68 841/220	NR Pirfenidone, prednisone, azathioprine, cyclophosphamide, cyclosporine, N-acetylcysteine 1-year disease progression	28 BMI < 27 NR	NR	Patients with BMI < 27 had a greater median annual rate of decline in FVC vs. placebo compared to those with BMI ≥ 27 (158, IQR 109–206 vs. 65, IQR 18–113, *p* = 0.007) No significant differences between patients with BMI <27 and ≥27 in absolute change in FVC (% predicted) vs. placebo (4.3, IQR 2.6–6.0 vs. 1.8, IQR 0.4–3.2, *p* = 0.37), absolute change in SGRQ (−2.6, IQR −5.7–0.6 vs. −0.4, IQR −3.2–2.3, *p* = 0.80), ≥1 acute exacerbation (HR = 0.65, 95% CI 0.34 to 1.26 vs. 0.65, 0.31 to 1.40, *p* = 0.96), and mortality (HR = 0.46, 95% CI 0.24 to 0.92 vs. 1.07, 0.52 to 2.19, *p* = 0.11)
Nakano A, 2020, Japan [45]	R	119 67 98/21	ATS/ERS/JRS/ALAT Pirfenidone, corticosteroids 7-year mortality	23 Relative decline in BMI in the first 6 months (%) NR	HR = 1.036, 95% CI 0.896–1.088, *p* = 0.163 Relative decline in FCV (% predicted), relative decline in ESM_CSA_	Significant correlation between relative decline in BMI and relative decline in ESM_CSA_ (r = 0.394, *p* < 0.001)
Tang F, 2020, USA [46]	P	1,061 68 841/220	NR Pirfenidone, prednisone, azathioprine, cyclophosphamide, cyclosporine, N-acetylcysteine 1-year acute exacerbation	28 Continuous variable NR	NR	No significant association between BMI and acute exacerbation in univariate Cox regression (HR = 0.958, 95% CI 0.906 to 1.010, *p*-value NR)
Zaman T, 2020, USA [47]	R	1,263 68 901/362	ATS/ERS/JRS/ALAT NR Disease progression over 3 years	29 BMI increase by a factor of 5 NR	NR	No significant association between BMI and progression in univariate Cox regression in the whole population (HR = 0.942, 95% CI 0.675 to 1.321, *p*-value NR) males (HR = 1.213, 95% CI 0.704 to 2.113, *p*-value NR) and females (HR = 0.821, 95% CI 0.538 to 1.242, *p*-value NR)
Kim HJ, 2021, USA [48]	R	1,002 70 747/255	ATS/ERS/JRS/ALAT NR Respiratory-related hospitalization within 2 years	29 Continuous variable NR	HR = 0.96, 95% CI 0.93 to 0.98, *p* < 0.001 Age, FVC (% predicted), O_2_ use at rest, pulmonary hypertension	NR
Sangani RG, 2021, USA [49]	R	138 76 83/55	ATS/ERS/JRS/ALAT Pirfenidone, nintedanib Mortality	NR BMI < 30 HT, HL, GORD, COPD, HF, OSA, DM, PH	NR	Mortality significantly higher in patients with BMI < 30 than in those with BMI ≥30 (34.8% vs. 20.4%, *p* = 0.018)
Suzuki Y, 2021, Japan [50]	R	208 72 176/32	ATS/ERS/JRS/ALAT Pirfenidone, nintedanib, immunosuppressants, N-acetylcysteine 5-year mortality	23 Continuous variable or BMI < 24.1 NR	*Continuous variable* HR = 0.920, 95% CI 0.847 to 0.996, *p* = 0.04 Age, sex, ESM_CSA_, FVC (% predicted), D_LCO_ (% predicted) *BMI < 24.1* HR = 1.673, 95% CI 1.063 to 2.709, *p* = 0.03 Age, sex, ESM_CSA_, FVC (% predicted)	NR
Uchida Y, 2021, Japan [51]	R	71 78 52/19	ATS/ERS/JRS/ALAT Nintedanib, prednisolone, tacrolimus Early nintedanib termination (at 6 months)	21 Continuous variable NR	HR = 0.862, 95% CI 0.715 to 1.040, *p* = 0.12 FVC (% predicted)	NR
Zinellu A, 2021, Italy [52]	R	82 72 73/9	ATS/ERS Pirfenidone, nintedanib 4-year mortality	27 Continuous variable HT, CAD, GORD, PH, COPD, OSA, AF	HR = 0.859, 95% CI 0.768 to 0.960, *p* = 0.007 Age, sex, smoking status, disease stage, antifibrotic drugs, aggregate index of systemic inflammation	NR
Jouneau S, 2022, France [53]	P	153 72 119/34	ATS/ERS/JRS/ALAT Pirfenidone, nintedanib, corticosteroids 1-year all-cause hospitalization, 1-year mortality	27 Continuous variable HT, CAD, CVA, AF	*Hospitalization* HR = 0.89, 95% CI 0.83 to 0.96, *p* = 0.003 GAP score, simple evaluation of food intake *Mortality* HR = 0.89, 95% CI 0.82 to 0.96, *p* = 0.003 GAP score, simple evaluation of food intake	Patients with BMI < 21 had a higher rate of acute exacerbation compared to those with BMI > 21 (73.1% vs. 41.7%, *p* = value NR)
Jouneau S, 2022, France [54]	P	1,604 67 1,374/230	ATS/ERS/JRS/ALAT Pirfenidone, interferon-γ-1b, lebrikizumab 1-year disease progression, 1-year, hospitalization, and 1-year mortality	30 Continuous variable NR	NR	Patients with baseline BMI < 25, 25–30, or ≥30 kg/m^2^ showed annualized change in (*p*-values NR): FVC (% predicted) of −6.6, −5.4, and −4.1, respectively D_LCO_ (% predicted) of −5.5, −5.0, and −4.0, respectively 6MWTD of −42.8, −32.5, and −30.5 m, respectively SGRQ total score of 5.8, 5.2, and 3.1, respectively and: Relative decline in percent predicted FVC ≥10% or death in 19%, 15.1% and 9.4%, respectively Any all-cause hospitalization in 23.8%, 25.4%, and 24.5%, respectively All-cause mortality in 6.7%, 7.9%, and 6.2%, respectively Any treatment-emergent serious adverse effect in 26.7%, 30.6%, and 27.0%, respectively
Yoo JW, 2022, South Korea [55]	R	445 67 335/110	ATS/ERS/JRS/ALAT Steroid, azathioprine, mycophenolate mofetil, cyclosporine 3-year acute exacerbation, 3-year mortality	24 Continuous variable NR	*Acute exacerbation* NR *Mortality* HR = 0.944, 95% CI 0.894 to 0.997, *p* = 0.037 Age, Charlson comorbidity index, FVC (% predicted), D_LCO_ (% predicted), 6MWT distance, 6MWT resting and lowest SpO_2_, disease progression, acute exacerbation	No significant association between BMI and acute exacerbation in univariate Cox regression (HR = 0.973, 95% CI 0.902 to 1.049, *p* = 0.470)
Zinellu A, 2022, Italy [56]	R	90 70 79/11	ATS/ERS Pirfenidone, nintedanib 4-year mortality	26 Continuous variable NR	HR = 0.82, 95% CI 0.71 to 0.95, *p* = 0.008 Age, sex, smoking status, treatment	AUC for BMI to predict mortality (0.702, 95% CI 0.596 to 0.794, *p* = 0.0001)

Legend: AF, atrial fibrillation; ALAT, Asociación Latinoamericana de Tórax; ATS, American Thoracic Society; AUC, area under the curve; BMI, body mass index; CAD, coronary artery disease; COPD, chronic obstructive pulmonary disease; CPI, composite physiologic index; CT, computed tomography; CVA, cerebrovascular disease; D_LCO_, diffusion capacity for carbon monoxide; DM, diabetes mellitus; ERS, European Respiratory Society; ESM_CSA_, cross-sectional area of elector spine muscles; ESM_MA_, muscle attenuation of elector spine muscles; F, female; FEV_1_, forced expiratory volume in the 1st second; FVC: forced vital capacity; GAP, gender age physiology; GORD, gastroesophageal reflux disease; HF, heart failure; HL, hyperlipidemia; HR, hazard ratio; HRCT, high-resolution computed tomography; HT, hypertension; IQR, interquartile range; JRS, Japanese Respiratory Society; M, male; mMRC, modified Medical Research Council dyspnea scale; NR, not reported; OP, osteoporosis; OSA, obstructive sleep apnea; P, prospective; PH, pulmonary hypertension; R, retrospective; SGRQ, St. George’s Respiratory Questionnaire; TLC, total lung capacity; 6MWT, six-minute walking test; 6MWTD, six-minute walking test distance.

**Table 2:** The Joanna Briggs Institute critical appraisal checklist.

Study	Were the Groups Comparable Other than the BMI?	Were the Same Criteria Used to Identify Cases and Controls?	Was Exposure Measured in a Valid and Reliable Way?	Was Exposure Similarly Measured in Cases and Controls?	Were Confounding Factors Identified?	Were Strategies to Deal with Confounders Stated?	Were Outcomes Assessed in a Valid, and Reliable Way for Cases and Controls?	Was the Exposure Period of Interest Long Enough to Be Meaningful?	Was Appropriate Statistical Analysis Used?	Risk of Bias
Alakhras M [21]	No	Yes	Yes	Yes	Yes	Yes	Yes	Yes	Yes	Low
Kondoh Y [22]	No	Yes	Yes	Yes	Yes	Yes	Yes	Yes	Yes	Low
Judge EP [23]	No	NR	NR	NR	No	No	Yes	Yes	Yes	High
Kim JH [24]	No	Yes	Yes	Yes	Yes	Yes	Yes	Yes	Yes	Low
Lee JS [25]	No	Yes	Yes	Yes	No	No	Yes	Yes	Yes	High
Mura M [26]	No	Yes	Yes	Yes	No	No	Yes	Yes	Yes	High
Kondoh Y [27]	No	Yes	Yes	Yes	No	No	Yes	Yes	Yes	High
Cao M [28]	No	Yes	Yes	Yes	No	No	Yes	Yes	Yes	High
Kishaba T [29]	No	Yes	Yes	Yes	Yes	Yes	Yes	Yes	Yes	Low
Nishiyama O [30]	No	Yes	Yes	Yes	No	No	Yes	Yes	Yes	High
Dotan Y [31]	No	Yes	Yes	Yes	No	No	Yes	Yes	Yes	High
Nishimoto K [32]	No	Yes	Yes	Yes	Yes	Yes	Yes	Yes	Yes	Low
Suzuki Y [33]	No	Yes	Yes	Yes	Yes	Yes	Yes	Yes	Yes	Low
Hanaka T [34]	No	Yes	Yes	Yes	No	No	Yes	Yes	Yes	High
Ikeda S [35]	No	Yes	Yes	Yes	Yes	Yes	Yes	Yes	Yes	Low
Kato M [36]	No	Yes	Yes	Yes	Yes	Yes	Yes	Yes	Yes	Low
Kono M [37]	No	Yes	Yes	Yes	No	No	Yes	Yes	Yes	High
Kulkarni T [38]	No	Yes	Yes	Yes	Yes	Yes	Yes	Yes	Yes	Low
Li B [39]	No	Yes	Yes	Yes	Yes	Yes	Yes	Yes	Yes	Low
Alhamad EH [40]	No	Yes	Yes	Yes	Yes	Yes	Yes	Yes	Yes	Low
Dotan Y [41]	No	Yes	Yes	Yes	No	No	Yes	Yes	Yes	High
Fang C [42]	No	Yes	Yes	Yes	No	No	Yes	Yes	Yes	High
Ikeda K [43]	No	Yes	Yes	Yes	Yes	Yes	Yes	Yes	Yes	Low
Jouneau S [44]	No	NR	NR	NR	No	No	Yes	Yes	Yes	High
Nakano A [45]	No	Yes	Yes	Yes	Yes	Yes	Yes	Yes	Yes	Low
Tang F [46]	No	NR	NR	NR	No	No	Yes	Yes	Yes	High
Zaman T [47]	No	Yes	Yes	Yes	No	No	Yes	Yes	Yes	High
Kim HJ [48]	No	Yes	Yes	Yes	Yes	Yes	Yes	Yes	Yes	Low
Sangani RG [49]	No	Yes	Yes	Yes	No	No	Yes	Yes	Yes	High
Suzuki Y [50]	No	Yes	Yes	Yes	Yes	Yes	Yes	Yes	Yes	Low
Uchida Y [51]	No	Yes	Yes	Yes	Yes	Yes	Yes	Yes	Yes	Low
Zinellu A [52]	No	Yes	Yes	Yes	Yes	Yes	Yes	Yes	Yes	Low
Jouneau S [53]	No	Yes	Yes	Yes	Yes	Yes	Yes	Yes	Yes	Low
Jouneau S [54]	No	Yes	Yes	Yes	No	No	Yes	Yes	Yes	High
Yoo JW [55]	No	Yes	Yes	Yes	Yes	Yes	Yes	Yes	Yes	Low
Zinellu A [56]	No	Yes	Yes	Yes	Yes	Yes	Yes	Yes	Yes	Low

Legend: NR, not reported.

## Data Availability

The datasets used and/or analyzed in this study are available from the corresponding author on reasonable request.

## Supplementary Materials

The following supporting information can be downloaded at: https://www.mdpi.com/article/10.3390/jcm12020498/s1, Table S1: PRISMA 2020 abstract checklist; Table S2: PRISMA 2020 manuscript checklist.
